# Effects of Aging in Combination with High-Fat or Ketogenic Diet on Skeletal Muscle Atrophy Following Denervation in C57BL/6J Mice

**DOI:** 10.3390/nu18030478

**Published:** 2026-02-01

**Authors:** Mantas Dirmontas, Petras Minderis, Aivaras Ratkevicius

**Affiliations:** 1 Department of Health Promotion and Rehabilitation, Lithuanian Sports University, LT-44221 Kaunas, Lithuania; mantas.dirmontas@stud.lsu.lt; 2 Institute of Sport Science and Innovations, Lithuanian Sports University, LT-44221 Kaunas, Lithuania; petras.minderis@lsu.lt; 3 Sports and Exercise Medicine Centre, Queen Mary University of London, London E1 4NS, UK

**Keywords:** muscle denervation, muscle atrophy, aging, high-fat diet, ketogenic diet

## Abstract

Objectives: The aim of this study was to investigate the effect of the interaction between aging and high-fat diet (HFD) or ketogenic diet (KD) on denervation-induced muscle atrophy. Methods: In this study, 6-, 19- and 27-month-old male mice were studied after 12 weeks’ exposure to a regular chow diet, RD (kcal distribution: 13% fat, 57% carbohydrate, 30% protein), HFD (kcal distribution: 60% fat, 20% carbohydrates, 20% protein), or KD (kcal distribution: 80% fat, <1% carbohydrates, 20% protein). Gastrocnemius (GAS) and soleus (SOL) muscles were left denervated during the last 6 weeks of this 12-week dietary intervention (*n* = 10 for each group). Results: Denervation-induced atrophy was greater (*p* < 0.001) in GAS compared to SOL. There were no differences between type 1 and type 2 muscle fiber atrophy in adult SOL muscle. Muscle atrophy did not depend on the diet and was greater in adult than old mice. Both HFD and KD feeding reduced IGF-1 levels (*p* < 0.01) in GAS muscle compared with the RD independently of age. Myostatin levels in GAS muscle increased (*p* < 0.01) with age independently of the diets. Conclusions: Denervation-induced muscle atrophy does not depend on dietary fat intake and proceeds at a slower rate in old mice compared to adult mice.

## 1. Introduction

Several neurologic and neuromuscular diseases cause denervation of skeletal muscles. Similarly, peripheral nerve trauma also causes muscle denervation, which is followed by muscle atrophy [1].

Muscle atrophy can be influenced by dietary factors. It appears that a high-fat diet (HFD, 45–60% kcal from fat) has a different effect on the neuromuscular system than a ketogenic diet (KD, 80% kcal from fat). An HFD dysregulates peripheral nerve lipidomes and impairs mitochondrial function [2]. There is also evidence that an HFD, especially when combined with denervation, reduces insulin signaling and glucose transport in skeletal muscle T-tubules [3]. An HFD can also increase spontaneous acetylcholine release and end-plate noise at the neuromuscular junction, which impairs muscle functioning [4]. An 8-day exposure to an HFD accelerated denervation-induced atrophy in oxidative SOL muscle compared to a regular chow diet, though the glycolytic extensor digitorum longus (EDL) muscle was less affected [5]. These effects contrast with findings on KD feeding which improved distal nerve morphology compared to a regular chow diet [6]. KD potentiated electrical stimulation-induced peripheral nerve regeneration and functional recovery [7]. A KD can also blunt atrophy signaling and preserve myofiber size during muscle unloading, though KD effects on denervated muscle have not been studied [8]. There is also evidence that a KD improves motor performance in mouse models of Alzheimer’s disease [9]. These findings suggest that KD feeding in contrast to HFD might reduce denervation-induced muscle atrophy.

Aging complicates neuromuscular adaptations to dietary factors [10]. IGF-1 is a growth factor that is involved in muscle mass maintenance [11]. Reduced plasma IGF-1 levels were observed in older men with smaller muscle mass compared to age-matched controls [12]. Initial increase in muscle IGF-1 expression is followed by a gradual decline after denervation which might reduce signaling through the IGF-PI3K-AKT-mTOR pathway, which plays a major role in stimulated protein synthesis [13]. Muscle inactivity might also promote myostatin expression, which can also interfere with signaling through this pathway as well as inhibit myogenesis [14,15]. Interestingly, an HFD was shown to suppress IGF-1 levels in skeletal muscles [16]. It would be important to investigate whether the positive effects of a KD on neuromuscular health are mediated by preservation of IGF-1 levels and suppression of myostatin expression.

There is a shortage of studies investigating the effect of the interaction between aging and dietary fat intake on muscle atrophy after denervation. The aim of our study was to compare the effects of a regular chow diet (RD), HFD, and KD on denervation-induced muscle atrophy in slow-contracting SOL muscle and GAS muscle which is predominated by type 2 fast-twitch muscle fibers in adult, mature, and old mice. We expected that aging and an HFD would reduce muscle IGF-1 levels, promote myostatin expression, and accelerate denervation-induced muscle atrophy. In contrast, a KD may not adversely affect anabolic signaling pathways and could therefore result in less severe muscle atrophy following denervation compared with an HFD. Our previous studies demonstrated that an HFD caused a significant impairment in glucose tolerance during the initial 6 weeks of feeding, with relatively minimal additional deterioration over the subsequent 6-week period [17]. Accordingly, the interval from week 6 to week 12 of HFD feeding provides an appropriate window to examine the effects of muscle denervation in mice that are well adapted to this diet.

## 2. Materials and Methods

### 2.1. Animals and Diets

This study was approved by the Lithuanian Republic Alimentary and Veterinary Public Office (Ref. G2-90, 2018). It was carried out at the animal research facility of Lithuanian Sports University. C57BL/6J mouse strain males were studied at 6, 19, and 27 months of age, classified as adult, mature, and old, respectively. Mice were housed in standard cages at 20–21 °C temperature and 40–60% humidity with the normal 12 h/12 h light/dark cycle reversed. Initially, animals were fed a regular chow diet or RD (kcal distribution: 13% fat, 57% carbohydrate, 30% protein (Joniškio Grudai, Joniškis, Lithuania), and received tap water ad libitum before being isolated in separate cages at the age of 3 months (adult group), 16 months (mature group), and 24 months (old group) for 12-week exposure to three different diets. This included an RD as a control diet, an HFD (kcal distribution: 60% fat, 20% carbohydrates, 20% protein (D12492, Research Diets, New Brunswick, NJ, USA), or KD (kcal distribution: 80% fat, <1% carbohydrates, 20% protein (D03022101, Research Diets)). Ten mice were randomly assigned to each diet within each age group.

### 2.2. Muscle Denervation

After 6 weeks’ exposure to different diets, mice underwent GAS and SOL denervation under aseptic conditions by transecting the sciatic nerve branches innervating GAS and SOL muscles at their entry into the muscle belly, followed by removal of a distal nerve segment (1–2 mm) to preclude reinnervation. This procedure causes atrophy of these two muscles as well as hypertrophy of the synergistic plantaris muscle [18]. Surgery was performed under anesthesia using subcutaneous injection of a mix of ketamine (100 mg kg^−1^) and xylazine (10 mg kg^−1^). Mice were also administered ketoprofen (2.5 mg·kg^−1^) as an analgesic before surgery, immediately after the procedure, and again 24 h later. Surgery was initiated only after the absence of a withdrawal response to a foot-pad pinch was confirmed. The operated hindlimb was shaved, and a skin incision was made using a scalpel. The underlying muscle fibers were carefully separated with scissors to expose the branches of the sciatic nerve. After resection of a segment of the nerve, the wound was closed with sutures. Denervation was considered successful if, after the 6-week intervention, the plantaris muscle mass in the denervated hindlimb was at least 10% greater than that of the control hindlimb, indicating compensatory overloading of this muscle.

### 2.3. Metabolic Measurements and Tissue Sampling

Body mass (BM) was measured weekly. During the final week, mice were fasted for ten hours prior to the glucose tolerance test (GTT). Mice subsequently received an intraperitoneal injection of glucose (2 g kg^−1^). Blood glucose levels were then measured using a glucometer (Glucocard X-mini plus, ARKRAY, Kyoto, Japan) in samples obtained from the tail vein at baseline (0 min) and at 15, 30, 60, 90, and 120 min after injection. Glucose area under the curve (AUC) during the test was evaluated as an index of glucose tolerance [19]. Mice were sacrificed by exposure to CO_2_ at the end of this study. Afterwards, GAS and SOL muscles were dissected, weighed (ABS 80-4, Kern & Sohn GmbH, Balingen-Frommern, Germany), and frozen at −70 °C for later analysis.

### 2.4. Muscle Analysis

IGF-1 and myostatin levels in GAS muscle were measured using a Mouse IGF-1 ELISA Kit (MBS175810, MyBioSource, San Diego, CA, USA) and Mouse Myostatin ELISA Kit (MBS010589, MyBioSource) with a plate reader (Spark 10 M, Tecan Group Ltd., Männedorf, Switzerland), respectively. A 20 μL muscle sample was used in this analysis. The muscle sample was prepared as follows. GAS muscle tissue (40–70 mg) was transferred to ice-cold phosphate-buffered saline (PBS, pH 7.4) at a ratio of 1 mg muscle to 10 uL PBS. The tissue was then homogenized for 5 min using scissors on ice (+4 °C) and stored at −80 °C. On a separate occasion, frozen homogenates were defrosted by shaking for 30 min and centrifuged at 13,000× *g* for 10 min at +4 °C. The supernatants were taken and stored at −80 °C for subsequent analysis.

### 2.5. Muscle Fiber Properties

The muscle fiber cross-sectional area (CSA) was assessed as in our previous studies [19]. Transverse muscle sections were cut at a thickness of 10 μm at −20 °C using a cryotome (CM1850UV, Leica, Wetzlar, Germany). These cross sections mounted on glass plates were then stained for myosin ATPase after pre-incubation at a pH of 4.34 as previously described [20]. Briefly, this procedure included five steps. Firstly, cross sections mounted on glass plates were preincubated for 5 min in Solution 1 (100 mM CH_3_COONa 100 mM KCl, pH 4.34). Secondly, the cross sections were transferred into Solution 2 (3 mM ATP, 100 mM C_2_H_5_NO_2_, 72 mM CaCl_2_, 100 mM NaCl, 100 mM NaOH, pH 9.4) and incubated for 30 min. Thirdly, the cross sections were transferred into Solution 3 (90 mM CaCl_2_) and incubated for 5 min. Fourthly, the cross sections were transferred into Solution 4 (154 mM CoCl_2_)) and incubated for 5 min. Fifthly, the cross sections were transferred into Solution 5 (160 mM (NH_4_)_2_SO_4_) for 1 min. Following each step, the plates containing the cross sections were rinsed three times with distilled water. Type 1 fibers stained in dark color and type 2 fibers were beige at the end of this procedure.

Microscopic images of the sections were then taken at four times magnification and ImageJ software (NIH—version 1.43, USA) was used for image analysis. The numbers of type 1 and 2 fibers were counted. The fiber CSA was assessed in 25% of the randomly selected fibers of each type.

### 2.6. Statistical Analysis

Statistical analyses were performed using Prism 10.0 and IBM SPSS Statistics (version 29). No animals or data points were excluded from the analysis when data were available. Measurements of IGF-1 and myostatin in the gastrocnemius (GAS) muscle were conducted in every second randomly selected muscle from each study group. Data normality was verified using the Shapiro–Wilk test. Analysis of variance (ANOVA) with Bonferroni’s post hoc test was used to assess effects of age (adult, mature, and old), diet (RD, HFD, and KD), denervation (control and denervated muscles), and the interaction between these factors. All post hoc tests were corrected for multiple comparisons. Pearson’s correlation coefficient was calculated to assess the strength of the association between variables. Linear regression analysis was also carried out. The level of significance was set at *p* < 0.05. All data are presented as means ± SD.

## 3. Results

### 3.1. Body Mass

**Figure 1:**
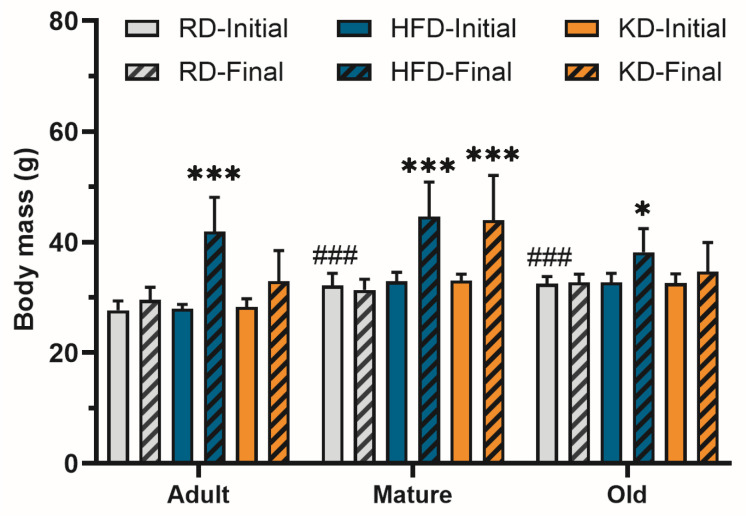
Initial and final body mass of mice fed regular diet (RD), high-fat diet (HFD), or ketogenic diet (KD) for 12 weeks, respectively. Data are shown as means ± SD (*n* = 10); * *p* < 0.05, *** *p* < 0.001 compared to the initial body mass of the same mice; ### *p* < 0.001 compared to RD-Initial group of adult mice.

Body mass data are presented in Figure 1. Initial body mass was greater in mature and old mice than in adult mice (*p* < 0.01), with no differences among diet groups within any age category. Final body mass was significantly influenced by diet (*p* < 0.001) and age (*p* = 0.002), with a significant diet × age interaction (*p* = 0.028). Across all age groups, high-fat diet (HFD) feeding resulted in greater body mass compared with regular diet (RD) (*p* < 0.01). Ketogenic diet (KD) feeding increased body mass relative to RD in the mature group (*p* < 0.001) but not in adult or old mice.

### 3.2. Glucose Tolerance

**Figure 2:**
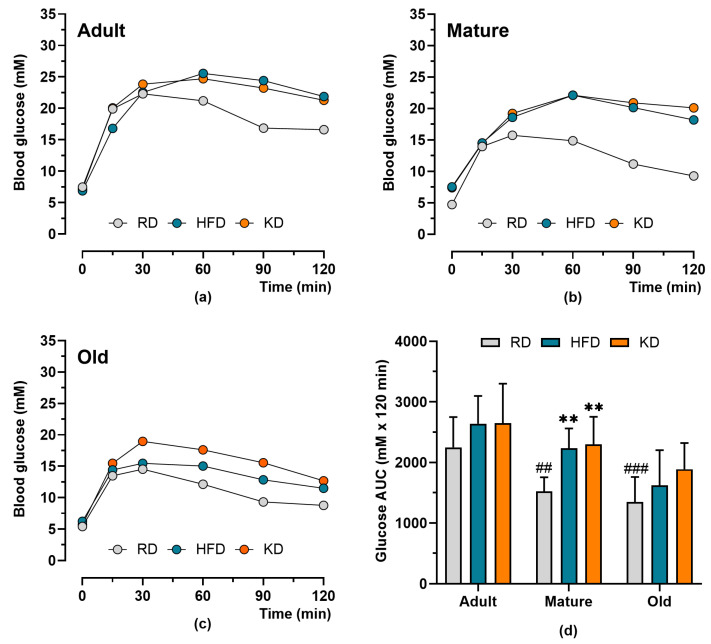
Blood glucose during the glucose tolerance tests in adult (**a**), mature (**b**), and old (**c**) mice fed regular diet (RD), high-fat diet (HFD), or ketogenic diet (KD) for 12 weeks. Glucose area under the curve (glucose AUC) is also shown for all age and diet groups (**d**). Data are presented as means ± SD (*n* = 8–10). ## *p* < 0.01, ### *p* < 0.01 compared to adult mice, ** *p* < 0.01 compared to RD in the same age group.

Glucose tolerance test data are shown in Figure 2. Blood glucose concentrations peaked 30–60 min after glucose administration and declined thereafter. This decline was attenuated in mice fed a high-fat diet (HFD) or ketogenic diet (KD) compared with those fed a regular diet (RD), particularly in adult and mature mice. Glucose AUC was significantly influenced by age (*p* < 0.001) and diet (*p* < 0.001), with no significant age × diet interaction (*p* = 0.547). Increasing age was associated with lower glucose AUC, whereas HFD and KD feeding resulted in higher glucose AUC values. The adverse effects of the HFD and KD on glucose tolerance were most pronounced in mature mice (*p* < 0.01).

### 3.3. Gastrocnemius and Soleus Mass

**Figure 3:**
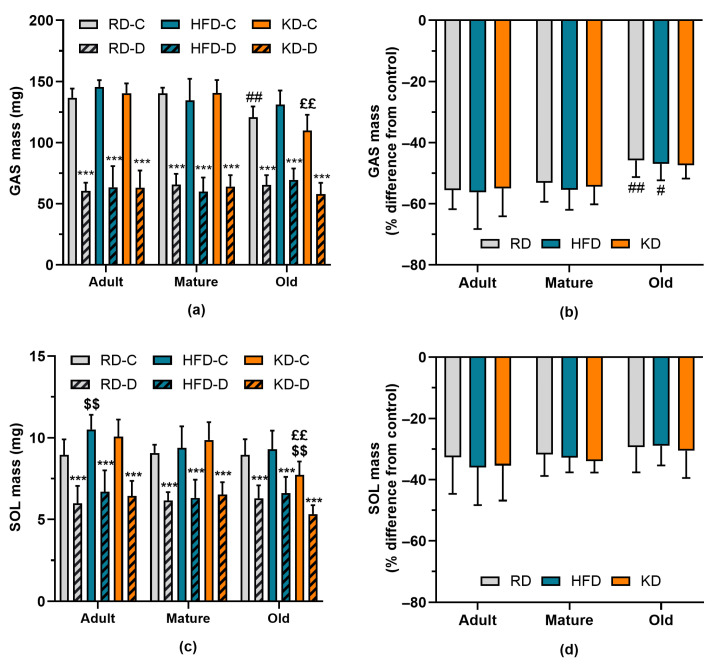
Gastrocnemius (GAS) and soleus (SOL) muscle mass were measured in control (C) and denervated (D) muscles from mice fed a regular diet (RD), high-fat diet (HFD), or ketogenic diet (KD) for 12 weeks. Panels (**a**,**c**) present the absolute muscle mass of GAS and SOL, respectively, whereas panels (**b**,**d**) show the percentage difference in muscle mass between denervated and control muscles for GAS and SOL. Data are means ± SD (*n* = 10); *** *p* < 0.001 compared to control (C) muscle, # *p* < 0.05, ## *p* < 0.01 compared to the respective adult mice on the same diet, $$ *p* < 0.01 compared to RD-C in the same age group, ££ *p* < 0.01 compared to HFD-C in the same age group.

Data on gastrocnemius (GAS) and soleus (SOL) muscle mass are presented in Figure 3. GAS mass declined with age (*p* < 0.001) and was significantly reduced by denervation (*p* < 0.001), with a significant age × denervation interaction (*p* < 0.001). In control muscles, GAS mass was lower in old mice fed a regular diet (RD) compared with adult mice (*p* < 0.01, Figure 3a). The ketogenic diet (KD) tended to further reduce control GAS mass when compared with the high-fat diet (HFD). Denervation caused a marked reduction in GAS mass; however, this denervation-induced atrophy was attenuated in old mice relative to adult mice (*p* < 0.01; Figure 3b). Control SOL mass was also negatively affected by age (*p* < 0.001), although the age-related decline was less pronounced than that observed in GAS. HFD increased control SOL mass compared with RD in adult mice, but this effect was not significant in mature or old mice. In contrast, KD appeared to exert a stronger negative effect on SOL than on GAS in old mice, with SOL mass reduced relative to both RD (*p* < 0.05) and HFD groups (*p* < 0.01). Similarly to GAS, denervation-induced atrophy of SOL tended to be less pronounced (*p* = 0.086) with increasing age of mice. Overall, muscle atrophy was significantly less severe in SOL than in GAS (*p* < 0.001), and age-related effects were correspondingly less marked in SOL.

### 3.4. Cross-Sectional Areas of Type 1 and 2 Muscle Fibers

**Figure 4:**
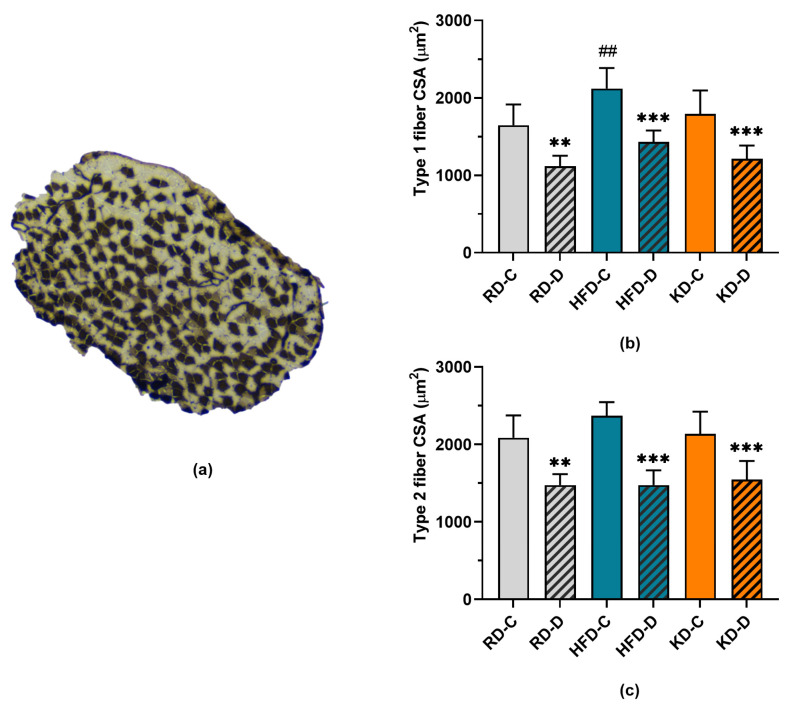
(**a**) Representative cross section of adult mouse soleus (SOL) muscle stained to distinguish type 1 (darkly stained) and type 2 muscle fibers. Panels (**b**,**c**) show the cross-sectional area (CSA) of type 1 and type 2 fibers, respectively, in control (C) and denervated (D) SOL muscles from adult mice fed a regular diet (RD), high-fat diet (HFD), or ketogenic diet (KD). Data are presented as means ± SD (*n* = 6). ## *p* < 0.01 vs. RD-C group; ** *p* < 0.01 and *** *p* < 0.001 vs. control (C) muscle.

We examined whether denervation-induced atrophy was fiber type-dependent using cross sections of adult soleus (SOL) muscle (Figure 4). Consistent with SOL muscle mass data (Figure 3D), HFD feeding increased in type 1 CSA compared to RD feeding (*p* < 0.003). In contrast, type 2 fiber CSA was not affected by the diet. Denervation induced a significant reduction in muscle fiber CSA (*p* < 0.001), and the magnitude of atrophy did not differ between fiber types or among diet groups.

### 3.5. Muscle Atrophy Versus Glucose Tolerance

**Figure 5:**
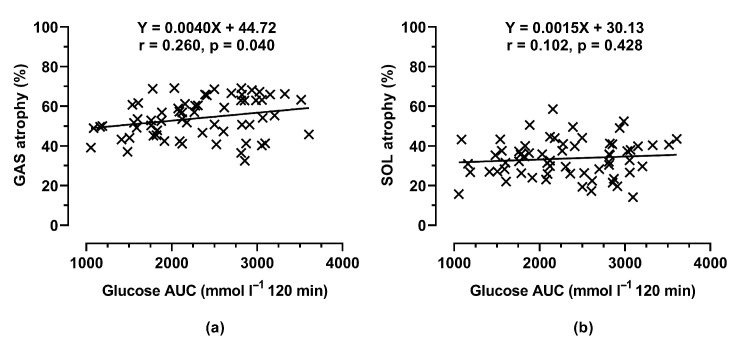
Relationship between glucose area under the curve (AUC) during the glucose tolerance test and muscle atrophy in (**a**) the gastrocnemius (GAS) and (**b**) the soleus (SOL). Scatter plots were generated using pooled data from all age groups and diet conditions. Pearson’s correlation coefficients (r) and the corresponding linear regression equations are shown.

Muscle atrophy rates may be influenced by metabolic health. The relationship between muscle atrophy and glucose tolerance, assessed by glucose area under the curve (AUC), is shown in Figure 5. A significant positive correlation was observed between glucose AUC and gastrocnemius (GAS) muscle atrophy (r = 0.260, *p* = 0.040), whereas no such association was detected in the soleus (SOL) muscle (r = 0.102, *p* = 0.428).

### 3.6. IGF-1 and Myostatin Levels in Gastrocnemius Muscle

**Figure 6:**
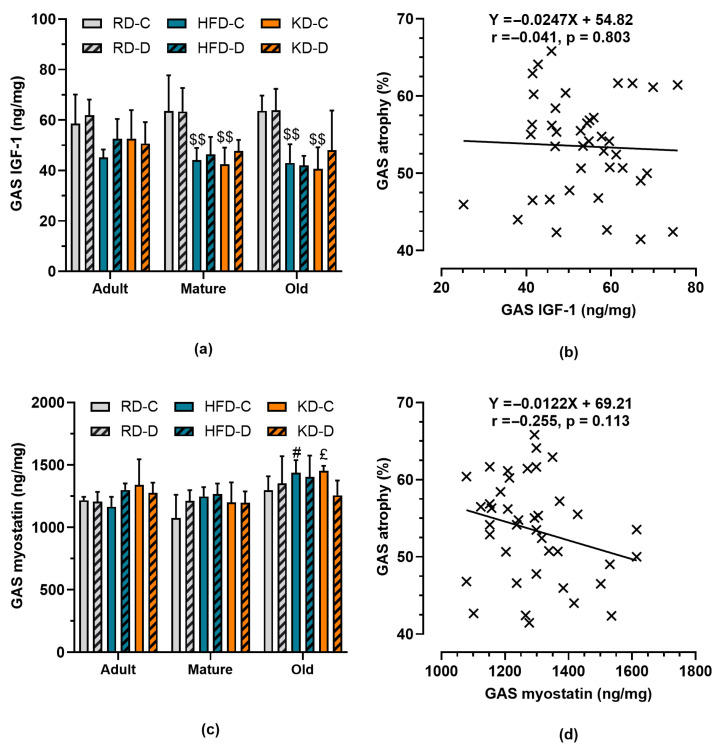
Gastrocnemius (GAS) muscle levels of (**a**) IGF-1 and (**c**) myostatin in control (C) and denervated (D) muscles from mice fed a regular diet (RD), high-fat diet (HFD), or ketogenic diet (KD) for 12 weeks. Data are presented as means ± SD (*n* = 4–5). $$ *p* < 0.01 vs. RD within the same age group; # *p* < 0.05 vs. adult mice fed HFD; £ *p* < 0.05 vs. mature mice fed KD. Panels (**b**,**d**) depict the relationships between IGF-1 and myostatin levels, respectively, and muscle atrophy, based on scatter plots generated from pooled data across all age groups and diets (*n* = 5–6 per group). Pearson’s correlation coefficients (r) and the corresponding linear regression equations are shown.

We examined changes in IGF-1 and myostatin levels in both control and denervated GAS muscles (Figure 6). Muscle IGF-1 levels were dependent on diet (*p* < 0.001) as HFD and KD feeding was associated with reduced muscle IGF-1 levels (*p* < 0.01) independently of age (*p* = 0.674). In contrast, myostatin levels increased (*p* < 0.001) with age independently of diet. There was no significant correlation between GAS atrophy and IGF-1 levels (r = −0.043, *p* = 0.801) or myostatin levels (r = −0.286, *p* = 0.108).

## 4. Discussion

The present study aimed to examine the combined effects of aging and high-fat diet feeding on denervation-induced muscle atrophy in C57BL/6J mice. Using GAS and SOL as representative glycolytic and oxidative muscles, respectively, we evaluated how age, diet, and molecular regulators including IGF-1 and myostatin influenced skeletal muscle mass after six weeks of denervation. This study helps to improve our understanding of how neuromuscular aging and dietary factors intersect in the context of denervation, which is relevant to peripheral nerve injury and motor neuron diseases such as amyotrophic lateral sclerosis (ALS). It is suggested that a high-carbohydrate diet may be more beneficial compared to a high-fat diet for ALS patients [21,22]. These recommendations contrast with opinions of other investigators emphasizing potential benefits of ketogenic diets for patients suffering from ALS and other neuromuscular diseases [23]. Our aim was to address this controversy by using a mouse model, allowing for a better control of diet compared to human nutrition studies.

Firstly, we found that denervation caused a profound loss of muscle mass, with greater atrophy in GAS compared to SOL. This is consistent with the vulnerability of fast-twitch muscles to inactivity [24,25]. Interestingly, atrophy did not differ between type 1 and type 2 fibers within SOL muscle, suggesting that oxidative versus glycolytic phenotype alone does not determine susceptibility to denervation-induced muscle wasting. Prior work showed that, after 14 days of denervation, oxidative muscles such as SOL are less resistant to atrophy compared to glycolytic EDL [5]. Our findings show that the difference between fiber types becomes less relevant if denervation is continued for longer periods of time and glycolytic muscles start showing a significant degree of muscle wasting. This may reflect the overriding impact of complete neuromuscular disconnection, which removes neural-derived trophic support across fiber types.

Secondly, old mice experienced slower rates of atrophy compared to adults. Similar results were reported in another study [26]. These findings are counterintuitive given the context of sarcopenia, where aging typically accelerates muscle loss [27]. Our results align, however, with reports that aged muscles exhibit blunted remodelling responses to both anabolic and catabolic stimuli [28]. In agreement with this line of reasoning, old mice show reduced skeletal muscle atrophy compared to adult mice after 48 h fasting [29]. Reduced plasticity in old muscle may therefore limit the rate of muscle atrophy. The smaller denervation-induced decline in old mice could also result from increased extracellular matrix accumulation, which may provide mechanical resistance to fiber shrinkage [30]. Clinically, this suggests that while aged muscles appear “protected” against rapid atrophy after denervation, they may simultaneously possess limited capacity for reinnervation and functional recovery [31,32].

Thirdly, dietary manipulation exerted surprisingly little effect on the magnitude of atrophy. There was only a weak association between glucose tolerance and muscle atrophy when data from all age groups and diets were pooled together. Neither the HFD nor the KD altered denervation-induced muscle loss compared to the RD, despite significant reductions in muscle IGF-1 in both fatty diets. In line with prior work, IGF-1 declined with high-fat feeding [8], yet this did not translate into measurable changes in the extent of atrophy. This disconnect suggests that local IGF-1 levels may not be the limiting factor in denervation-induced muscle loss. Martin et al. [13] reported a transient increase followed by a decline in IGF-1 after denervation, indicating a complex time-dependent regulation. The lack of correlation between IGF-1 and atrophy observed here supports the view that systemic factors and neural trophic signals outweigh local growth factor availability in this context.

Interestingly, the KD exerted a negative effect on muscle mass in old mice. KD feeding exacerbated age-related GAS wasting compared with RD. While a KD has been reported to enhance nerve regeneration [7] and protect against muscle wasting in unloading models [9], its interaction with aging appears detrimental. This observation contrasts with KD studies showing preserved muscle function in aged mice fed KD throughout their lifespan [33]. However, Roberts et al. [33] carried out a KD under conditions of fixed energy intake of 11.2 kcal per day, which is likely to induce caloric restriction. This shows context, age, and feeding regimen critically determine KD outcomes.

Fourthly, myostatin levels increased with age, independently of diet. Myostatin, a TGF-β family member, is a well-known negative regulator of muscle mass [17,18]. Age-associated elevation of myostatin has been implicated in sarcopenia and reduced regenerative potential [34]. However, in our study, myostatin levels did not correlate with atrophy. Future studies using myostatin blockade could clarify its precise role in muscle mass maintenance during denervation.

From a translational perspective, our results caution against assuming that dietary fat manipulation alone can mitigate muscle atrophy after denervation. While ketogenic interventions may support nerve regeneration [6,8], their capacity to directly attenuate muscle wasting after denervation appears limited.

Several limitations of our study warrant mention. Firstly, only male mice were studied; sex differences in muscle aging and diet response are well recognized and should be investigated. Secondly, denervation was studied over six weeks, which may not capture chronic adaptations or late regenerative processes. Thirdly, food intake was not controlled, which may confound the findings. Finally, while IGF-1 and myostatin were measured, other pathways such as Akt/mTOR, FoxO, or inflammatory signaling were not examined but may provide mechanistic insights.

## 5. Conclusions

Our study demonstrates that denervation-induced skeletal muscle atrophy is more pronounced in fast-twitch gastrocnemius than slow-twitch soleus, and attenuated in old mice compared to adults. High-fat and ketogenic diets reduced IGF-1 but did not alter denervation-induced atrophy, though ketogenic diet exacerbated muscle wasting in old age. Aging was associated with elevated myostatin, yet neither IGF-1 nor myostatin predicted muscle loss. Age, however, modifies both the rate of atrophy and the response to ketogenic diet feeding.

## Data Availability

The data that support the findings of this study are available from the corresponding author upon reasonable request.

## Abbreviations

The following abbreviations are used in this manuscript:

RD	Regular diet
HFD	High-fat diet
KD	Ketogenic diet
GAS	Gastrocnemius muscle
SOL	Soleus muscle
ALS	Amyotrophic lateral sclerosis
NMJ	Neuromuscular junction
EDL	Extensor digitorum longus muscle
PL	Plantaris muscle
CSA	Cross-sectional area
BM	Body mass
AUC	Area under the curve
C	Control
D	Denervated
